# A New Dusts Sensor for Cultural Heritage Applications Based on Image Processing

**DOI:** 10.3390/s140609813

**Published:** 2014-06-04

**Authors:** Andrea Proietti, Fabio Leccese, Maurizio Caciotta, Fabio Morresi, Ulderico Santamaria, Carmela Malomo

**Affiliations:** 1 Department of Information Engineering, Electronics and Telecommunications (DIET), University of Rome “La Sapienza”, Via Eudossiana 18, 00184 Rome, Italy; E-Mail: andrea.proietti@uniroma1.it; 2 Science Department, University of “Roma Tre”, via della Vasca Navale 84, 00146 Rome, Italy; E-Mail: caciotta@uniroma3.it; 3 Vatican Museums, viale Vaticano, 00165, Roma, Italy; E-Mails: fabio.morresi@gmail.com (F.M.); santamaria@unitus.it (U.S.); 4 ProArt s.r.l., via Boccioni n.5, 00100, Rome, Italy; E-Mail: c.malomo@libero.it

**Keywords:** dust detection, image processing, cultural heritage, artworks preservation

## Abstract

In this paper, we propose a new sensor for the detection and analysis of dusts (seen as powders and fibers) in indoor environments, especially designed for applications in the field of Cultural Heritage or in other contexts where the presence of dust requires special care (surgery, clean rooms, *etc.*). The presented system relies on image processing techniques (enhancement, noise reduction, segmentation, metrics analysis) and it allows obtaining both qualitative and quantitative information on the accumulation of dust. This information aims to identify the geometric and topological features of the elements of the deposit. The curators can use this information in order to design suitable prevention and maintenance actions for objects and environments. The sensor consists of simple and relatively cheap tools, based on a high-resolution image acquisition system, a preprocessing software to improve the captured image and an analysis algorithm for the feature extraction and the classification of the elements of the dust deposit. We carried out some tests in order to validate the system operation. These tests were performed within the Sistine Chapel in the Vatican Museums, showing the good performance of the proposed sensor in terms of execution time and classification accuracy.

## 1. Introduction

The science of cultural heritage conservation is a wide and extremely structured topic, involving many fields of applied scientific research. This mainly depend on the strategies of preventive conservation, whose characteristic is the multidisciplinary. Among all these strategies, the active control of environmental parameters as air circulation, temperature, relative humidity, pollution and solar radiation, plays an essential role [1]. Nowadays, a suitable environment for the fully preservation of artworks is not strictly defined and there are not fast and standard solutions to develop *ad-hoc* applications for museum environments. In particular, it is not possible to apply standard schemes for air quality and climate control, due to the different types of artworks in the museums. In order to design such systems, it is necessary to consider at least the three principal components of a museum system: the museum itself (a mixture of building, environment and location), the artworks and the visitors. Each one can be a source of problems, as described in the following.

(1) The museum
  - It is located in a specific climate and environmental context, and then it is impossible to design a standard strategy for any set-up.
  - The building may have constraints concerning the implementation of substantial tasks, especially when it is a cultural asset itself, as frequently happens.
(2) The artworks
  - They are made of different materials, then they require considering all the physical, chemical and biological phenomena that may occur in the particular conditions and all the possible interactions with the tools used for exhibition and conservation.
  - Each artwork has a previous environmental history, which affects the material degradation.
(3) The users
  - They require suitable accessibility and enjoyment conditions, which can create some conservation issues. Each user modifies the conditions of the exposition environments, introducing dust and other pollutants from the outside, altering temperature and humidity as well.
  - They require continuity in the museum services, imposing the minimization of works designed to improve the quality of the museum environment.

All the previous remarks aim to correctly design an air quality and microclimate control system, which is the key element affecting the production, the transport and the accumulation of the dust. This can affect artworks and objects exhibited in museum environments, especially by its accumulation. The damages caused by the accumulation of dust affect several types of objects and materials, sometimes with an irreversible impact. The dust impacts on the conservation status of objects and surfaces, on the quality level of their exposure and, therefore, on the experience of the visitor. Therefore, the management of the powder is critical to the preservation of cultural heritage and it is a problem of considerable scientific interest by more than one century [2].

Current techniques allow the monitoring and the analysis of the dust by using onerous methodologies, both from the economic viewpoint (very expensive and brittle instrumentation) and the technological viewpoint (bulky equipment and composite measurements). These especially rely on Energy Dispersive X-ray spectroscopy, X-ray fluorescence spectroscopy, scanning electron microscopy, optic microscopy, thermography, filters [3–12]. This equipment allows obtaining detailed information about the shape and the dimensions of the dust elements, on its chemical composition, on the bacterial load, and about other physical features. In most of the contexts, where the problem is not so critical, but it is nonetheless significant, the design of the ventilation, filtering and dust suppression systems requires only information regarding the amount, the shape and the size of the elements of the dust deposit, as will be explained in the following.

In this paper, we propose a new approach for dust detection and analysis based on the acquisition and preprocessing of images of dust accumulations, and the subsequent application of image processing techniques in order to obtain the previously mentioned information about the amount, the shape and the size of the elements of the dust deposit. In Section 2, we present a brief overview of the issues relating to the conservation of the artworks, paying particular attention to the problems related to dust. In Section 3, we describe the device used to capture the dust accumulations images and we analyse the preprocessing tasks aimed at preparing images for the application of our approach. Moreover, we describe the proposed method for the analysis of dust accumulation based on the captured and preprocessed images. Finally, in Section 4, we report the test activities, describing the experimental environment (the “Cantoria” of the Sistine Chapel, at the Vatican Museums in Rome, Italy) and showing some results to illustrate the capability of our approach in the analysis of the dust elements. Moreover, we present some correlations between the obtained results and the experimental site.

## 2. Artworks Preservation Issues

An artwork, likewise historical objects exposed in a museum, is a “material evidence” of the path of civil and cultural growth of the Human Being and it should be always in optimal conditions of preservation. Unluckily, though slow in time, all objects suffer from deterioration: e.g., metals undergo a corrosion process, fungi and bacteria attack paintings, papers, woods and tissues. Generically, it is possible to affirm that are activated some processes on the objects that alter the original state, undermining their integrity. Clearly, this depends on the materials composing the object and on the interactions among these materials, on the thermo-hygrometric conditions, and on the air quality of the environment.

Paintings, as artworks, are extremely sensitive to many agents that might cause their degradation. In a museum environment, the most important are:

(1) Carbon dioxide. It strongly depends on anthropic activities and it can disjoint limestone and frescoes. In the presence of water, carbon dioxide reacts with the calcium carbonate producing carbonic acid, H_2_CO_3_, soluble in water by the following reaction:
  (1) $${\text{CaCO}}_{3}+{\text{H}}_{2}\text{O}+{\text{CO}}_{2}↔\text{Ca}{\left({\text{HCO}}_{3}\right)}_{2}$$
  The greater the concentration of carbon dioxide in solution, the greater is the formation of soluble bicarbonate, while the decrease in the concentration of CO_2_ leads to recrystallization of the carbonate, determining both the formation of carbonate veils on the artworks surface, especially on calcareous stones and frescoes, and their disintegration.
(2) Sulphur derivatives. Sulphuric acid corrodes organic materials and metals. Sulphur dioxide can transform in dust many materials such as silk, wool, skin, vellum, *etc*. Sulphur dioxide is present in high quantities in the atmosphere, as a result of the combustion of fossil fuels. It is particularly reactive in the atmosphere where, combined with molecules of oxygen and steam, it leads to the formation of sulphur trioxide, SO_3_, and then to the formation of sulphuric acid, H_2_SO_4_, and sulphates, according to the following reactions:
  (2) $$\begin{array}{c} \text{S}+{\text{SO}}_{2}\to {\text{SO}}_{2}\\ 2{\text{SO}}_{2}+{\text{O}}_{2}\to 2{\text{SO}}_{3}\\ 2{\text{SO}}_{3}+{\text{H}}_{2}\text{O}\to {\text{H}}_{2}{\text{SO}}_{4} \end{array}$$
  All these molecules act on the artworks and they became particularly harmful in combination with ultraviolet rays (UV). However, the most dangerous is sulphuric acid, which is highly corrosive when it falls on the materials' surface. It acts on materials such as gesso, limestone, marble, frescoes and alkaline sandstone creating sulphation of calcium carbonate, causing a risk of detachment in the presence of liquid water.
(3) Ozone. The principal reaction starts from the nitrogen dioxide, NO_2_, which is a product of the combustions and it is photo-dissociated by the UV rays, according to the following reaction:
  (3) $${\text{NO}}_{2}+\text{UV}\to \text{NO}+\text{O}$$
  The monoatomic oxygen reacts with oxygen present in the air forming ozone:
  (4) $$O_{2}+O\to O_{3}$$
  Then, the ozone reacts again with nitrogen monoxide:
  (5) $$NO+O_{3}\to NO_{2}+O_{2}$$
  This reaction cycle can produce ozone concentrations up to 0.4 ppm against the 0.02/0.05 ppm of unpolluted air. Ozone is one of the known strongest oxidants and it is toxic for humans because at concentrations above to 0.08 ppm it can irritate the eyes and respiratory system, while at concentrations above to 0.4 ppm it tends to reduce lung functions. As regards the effect on materials, the oxidizing properties of ozone lead to brittle rubbers, to faded tissues, destruction of unsaturated organic compounds, such as skins or natural pigments, and it accelerates the sulfurization of copper and silver.
(4) Halogen compounds. They are due to some industrial and artisanal works and they are essentially fluorides, chlorides and the derived acids that, even in small concentration, are particularly harmful for artworks, producing oxidation and corrosion of metals, dimming of glasses, and decomposition of ceramics, terracotta, and siliceous stones.
(5) Volatile organic compounds (VOC). They can be alkanes, cycloalkanes, aromatic hydrocarbons and chlorinated aldehydes. They are due to many substances commonly used in many contexts as solvents, deodorants, insecticides, glues, paints, cleaning products, floor waxes, *etc.*, but also they derive from combustion, tobacco smoke, human metabolism and from conditioning plants. Even if present in low concentrations, VOCs are particularly harmful for ceramics or materials based on calcium, such as shells or corals, which can be stained, discoloured, and made brittle. Moreover, they may increase the occurrence of surface efflorescence. Moreover, VOCs can corrode and oxidize metals, which can produce stained, discoloured, weak and brittle synthetic materials.
(6) Biological contaminants. They are divided in microorganisms such as protozoa, fungi and bacteria; insects such as mites and spiders; biological materials such as animal droppings or fragments of exoskeletons or scurf; and vegetal organic materials such as pollens. Among these contaminants, the microorganisms are the most harmful for artworks. They find suitable growth conditions in wet environments, for example due to surface condensation phenomena. Microorganisms affect paper, paintings on canvas, wood, photographs and other cellulose materials with macroscopic effects. Initially, they form stains of various colours, then, over time, the material loses consistency and disintegrates. The tissues are irreversibly stained, lose consistency and pierce, the skins and the plastics are mottled and brittle, while papyri better resist their actions because the original manufacture process involves the use of cedar oil that reduces the hygroscopicity of the material.
(7) Airborne particulates. This consists of particulates and filiform materials in suspension resulting from stationary combustion plants, traffic, industrial sources and visitors. Dust, soot, residues of tobacco smokes, broken textile fibres are relevant issues for artwork preservation, because under particular conditions they can create acids thanks to their high catalytic ability. Moreover, the accumulation of particles can cause fouling and deteriorations of the surface of materials [13]. Finally, dusts can bring biological contaminants, which may be dangerous for the artworks as previously described.

Among all these agents, dust (in the form of particulates and fibres) can be especially negative. In particular, dust can cause both tangible and intangible damages. The first relate to the visual result of a dusty object, *i.e.*, difficulties in vision or alteration of appearance. The latter concern the physical-chemical corruption of the objects, especially due to the onset of:

- Corrosion, due to the mineral particles or acids able to alter chemically the surfaces.
- Biological contamination, due to pollen, epidermal cells, fragments of insects and other organic material transported by the dust.
- Humidification, due to the hygroscopic capability of the dust to attract and retain water on the surface of objects, contributing to the formation of spots and stains, as well as to the two just described issues.

These problems are interrelated and frequently feed off each other. The accumulation of dust, in fact, may create accumulations of water that feed the growth and proliferation of microorganisms, which themselves may die and spread to other objects as contaminants [14]. The humidification and subsequent dehydration of the dust deposits, produced by the natural mechanisms of evaporation and drying, may cause the cementation of particulates on the involved surfaces. Additional factors that favour the cementation of dust on the artworks are the moisture and the annual period in which the phenomenon occurs, as well as other micro-climatic conditions [15]. Rugosity and electrostatic forces similarly favour the superficial adhesion. Furthermore, the dust accumulation mechanism even depends on the surface typology, on the transportation mechanism, on the climate and microclimate.

Dust generally consists of a mixture of components coming from the outdoors (road and soil dust, plants, insect fragments) or that are already located indoors (clothing or carpets fibres, skin fragments, food particles). These components may arise from a variety of sources. In particular, one of the leading causes of dust in indoor public spaces, such as museums, concerns the visitors. They can provide dust contributions through their shoes, their clothing and their bodies [16]. These contributions rely mainly on coarse particles (mostly transported from the outside) and fibres (principally generated from clothing). The latter commonly represent only about 3% of the deposit of dust in a museum environment, but being larger, and thus more visible, greatly affect the deposit at a visual level [17]. Some studies have demonstrated that, both in free atmosphere and inside museums [18], during the opening time there is a considerable increment of the fine particles with a diameter less of 1 mm and, during the night hours, these particles quickly sediment by gravitation. As suggested in [1], the size parameter does not have a strict scientific classification and, for our purposes, we classify the elements by size as:

- Fine, with diameter within the range from 0.05 to 1 mm, whose behaviour is comparable to that of gases;
- Coarse, with a diameter larger than 1 mm.

The most common traditional preventive measure against dust deposits is the use of glass cases (or in other transparent materials), which involve significant deployment, management and maintenance costs. Alternative prevention measures rely on the use of barriers or obligatory paths, which affect the usability of the exposure and the quality of the visit, providing a spatial separation of objects from the visitors. Moreover, suitable carpets placed at the entrance of the museum environments can capture the dirt transported from the outside. They undergo an appropriate process for exhaustive cleaning before opening to the visitors [19].

The traditional corrective measures almost always rely on periodical tasks of ordinary and extraordinary cleaning of the artworks and environments, which can preclude environment access by visitors or involve the partial closure of visiting areas and access to specific objects. Sometimes, these cleaning tasks can even worsen the problem, especially if they involve unsuitable methods, such as dusting, scrubbing or unfiltered vacuum cleaning, causing the spread of dust and surface abrasion. Moreover, the cleaning and maintenance activities are sometimes impractical, for example when they involve very fragile artworks. Even the use of forced ventilation systems, such as fans, or the presence of windows that can generate air currents, may worsen the problem, facilitating the transport processes and the propagation of dust. All these corrective measures are generally very expensive and require the use of several workers [19]. In addition, the planning of such tasks depends on the conservation criteria and the needs related to the visitors. Lastly, note that these measures are often subject to debate and discussion, especially because the individual museum authorities have a different understanding of the dust problem [20,21], as well as visitors [22,23].

The kind of material making up the dust plays a fundamental role in the development of the previously mentioned degradation processes and, hence, in the choice of the possible countermeasures as well. In particular, the design of air conditioning, ventilation and filtering systems should rely on objective data and analysis conducted on the powder, in order to identify its physical characteristics in terms of component materials. Furthermore, these analyses may provide useful information for differently conceiving the enjoyment of the museum and the artworks, such as limiting the number of visitors or introducing suitable showcases. As suggested in [24], the kind of material can be often deduced by the shape and the size of the dust element. For example, a filiform structure is related with textile fibres or hair, round structures may be dandruff or soil particulates and polygonal shapes could give an indication of pieces of plaster.

Currently, this kind of analysis is performed manually and they consist of sampling and analysis campaigns, useful for properly planning the countermeasures, in terms of costs and employed resources as well. Sampling and analysis of dust do not yet provide national or international standards, even if several methods have been developed for the monitoring of dust deposits and for their classification [25,26]. These often use just size or percentage covering as classification parameter or they rely on a microscopic photographic comparison. Furthermore, different types of sensors and devices have been developed for monitoring and analysis of the powder, but most of them detect only some data, such as concentration, opacity, and others [27–29]. These tools are often too expensive, using complex methodologies and thus preventing their frequent use. Other commercial dust sensors are developed to correct artefacts in digital cameras [30], used in automatic vacuum cleaners [31], or developed for particular applications such as the determination of dust in extra-terrestrial planets [32]. On the other hand, most of the researches carried out on the dust effects in Cultural Heritage do not involve new technologies, which would allow to automatically recognizing the dust [33]. In [34] the researchers employed physical sampling of the particulates, using an appropriate adherent material by which to collect dust deposits, in order to design transparent barriers for splitting the exhibition environment from the one dedicated to transit of the visitors. This reduced the influence of the flow of visitors on the deposit of dust, but it did not eliminate the problem at its source, as well as cause a significant impact on the quality of the visit. Another system [35] relies on the measurement of the light reflectance variation of the surfaces due to the deposits of dust. These methods require the use of special equipment and, in addition, address the issue only from the visual point of view. In [36] the researchers use similar methods for the collection of samples, using adhesive materials to capture the particulates deposited on the ground. The analysis of samples consists of the acquisition of images with an optical microscope. Subsequently, a computer processed them in order to extract information on the number of particles and on the area of each particle.

Our purpose is to propose an innovative method for the analysis of powder, which should allow getting more complete information concerning the amount of elements in the deposit of dust, their size in terms of covered area, their shape (distinguishing between particles and fibres), and the speed of accumulation of dust deposit. Moreover, these targets should be achieved with cheap and user-friendly methods and they should be non-invasive in the museum exhibition and for the user experience. In [37] we presented a starting point for the approach presented in this paper, analysing the basic idea and its potential for the analysis of dust in the Cultural Heritage context. In this paper, we present an advanced automatic solution that meets the previously mentioned requirements, showing the key elements and describing the stages of testing and experimentation.

## 3. Materials and Methods

In this section, after a brief introduction on the capturing device, we describe the image processing algorithm for the dust analysis, dividing it in the preprocessing and in the classification steps.

### 3.1. The Capturing Device

The image acquisition system consists of a standard desktop PC connected to an optical sensor. As described in the following, the control software allows capturing an image at customizable time interval and it saves the information in an uncompressed JPEG file.

**Figure 1.**
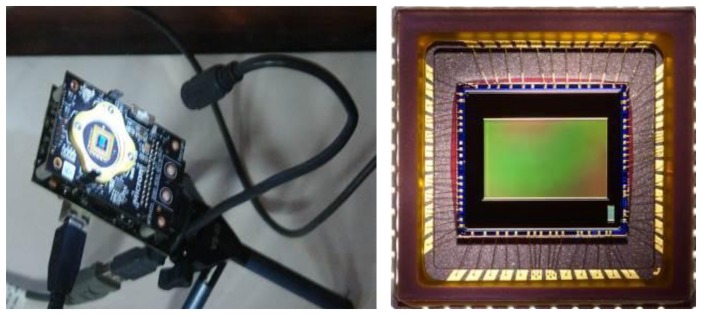
Micron MT9V032 with the LED lamp both connected by USB and a detail of the CMOS sensor, where the dust deposition occurs.

The device used is the Micron MT9V032 [38] shown in Figure 1. It is an optical sensor with a 8.47 mm diagonal based on CMOS technology and it is mainly used for surveillance needs, both for internal and external use. Its most interesting characteristic is the ability to work efficiently under a very wide range of lighting conditions, while maintaining a high frame rate (up to 60 fps). It is able to provide a resolved image both under conditions of low brightness (lower than 0.1 lux) and in case of direct solar light. The MT9V032 can efficiently work over a wide temperature range (303.15 K/343.15 K) and can work up to 393.15 K.

**Figure 2.**
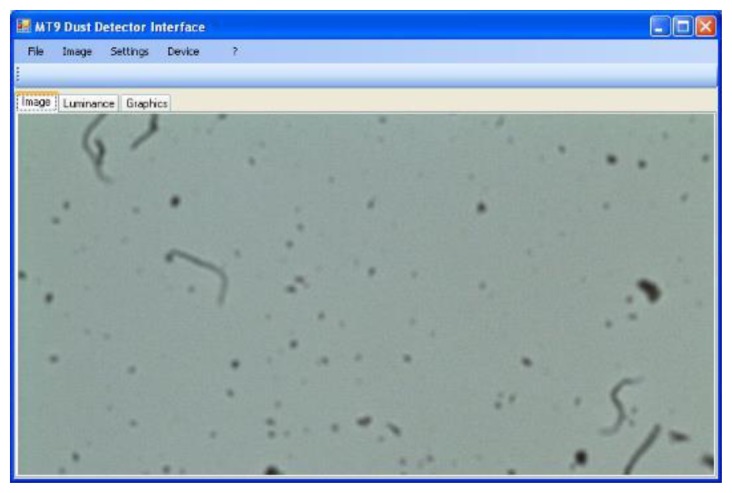
A screenshot of the MT9 Dust Detector Interface GUI.

In order to allow the direct accumulation of dust on the CMOS sensor, we removed the lens and the optical equipment of the device. The images obtained by this configuration show a continuous flickering, typically due to neon or incandescent lamps. In order to avoid this problem, it is possible to adjust the integration time to values multiple of the lighting frequency (50 Hz) or using a continuous light without oscillations, as LED. Hence, the system require the use of a USB LED lamp in order to have a stable image in any lighting condition and to improve the contrast. Note that the use of a 250 mW LED lamp does not create thermal effects. An *ad hoc* developed interface in the Microsoft environment allowed us to automatically acquire and elaborate the frames. The interface called MT9 Dust Detector Interface has three modules:

- The graphical user interface developed in C# (Figure 2);
- A DCOM component (provided by Micron) and managed in C#;
- A module for the image elaboration totally written in C++.

The interface between sensor and PC has been equipped with a timer section able to acquire the frame at prefixed time intervals. The lengths of the time interval between two different frame captures is customizable by the graphical user interface. The last frame acquired appears in the main window of the program. The images are then stored in uncompressed JPEG format, labelled with the acquisition timestamp.

### 3.2. The Preprocessing Steps

**Figure 3.**
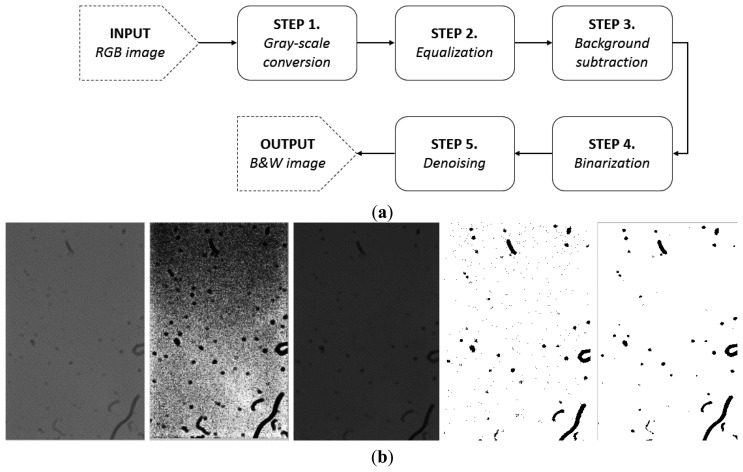
Preprocessing flowchart (**a**) and images resulting by each preprocessing steps (**b**).

The presented acquisition system imposes some limitations on the captured images. These depend especially on three factors: the focus, the lighting and the noise. As previously mentioned, removing the optical section causes the absence of focus in the acquired image. Furthermore, the lighting conditions imposes a brightness gradient and some residual flickering contribution on the captured image. Finally, the sensitivity of the sensor and the acquiring conditions cause noise, mainly in the form of spread dots. Hence, the obtained images require some preprocessing steps (Figure 3), as described in the following:

(1) Each captured frame is an RGB colour image, which requires a conversion into the grey-scale colour space in order to reduce the computational effort.
(2) The grey-scale image pass through a software equalization in order to obtain a contrast improvement and to enhance the image profile.
(3) The equalized images undergoes a removal of the brightness gradient shows in the image background (especially due to the use of the aforementioned LED lighting). This operation consists of the application of a low pass filter to isolate the background. Then, the original image undergoes a subtraction of this background, allowing avoiding this problem. A critical parameter of this step is the cut-off frequency. The choice of this parameter relies on the analysis of the amplitude spectrum of the Fourier transform of the image, since the background (*i.e.*, a gradient) depend on the low frequency contributions.
(4) The image resulting from the subtraction undergoes a binary conversion using a function based on the Otsu method [39]. This involves a thresholding to the grayscale image in order to convert it in a monochromatic image. This operation also aids in the elimination of noise.
(5) Finally, the binarized image is filtered with a median filter (whose square mask size is of 3 pixels) in order to reduce the residual noise (mostly spread dots, *i.e.*, individual pixels considered as noise).

In conclusion, the result of these steps is a cleaner image, where the dust consists of null values (black), while the neat results in unitary values (white).

Based on the acquired images and on the preprocessing results, it is possible to draw an immediate conclusion about the analysis and understanding of the dust accumulation phenomenon. This concerns the degree of dirtying of the image: it is possible to calculate the dirty image percentage by reversing the image in the form of white on black (white pixel for powder and black for clean) and summing the unitary contributions. This value is useful for qualitatively understand how quickly dust accumulates on the surfaces. For the quantitative analysis, it is possible to follow a path based on image processing tools, as described in the following.

### 3.3. The Analysis and Classification Algorithm

The proposed approach for the analysis of the images resulting from the preprocessing relies on classical image processing tools. It aims to provide two types of information: the shape of each element of the dust deposit and its size spectrum (in terms of area occupied by each element). This approach relies on an iterative structure which scans the reversed image (white on black), in order to identify and analyse the individual elements of dust. The algorithm builds a scanning p-square window, which is scrolled horizontally (pixel by pixel) from the upper left for all rows of the image; p is the length of the square side. For each position of the scanning window, three conditions are checked:

- The scanning window is not empty—the algorithm proceeds only if this condition is met, preventing by launch the processing with empty windows, gaining in terms of execution speed.
- The scanning window “completely contains the content”—the algorithm proceeds only if this condition is met, *i.e.*, only if the window contains the powder inside or, in other words, only if the edge of the window does not intersect filled pixels. This is useful to prevent misjudgements (e.g., due to partial coverage of the window on a filiform element, which would lead to exchange it for a granular element).
- The content is larger than a pixel—the algorithm proceeds only if the window contains more than one filled pixel; this prevents the execution on individual pixels, assuming them as noise.

Note that the system runs with value of p greater than 4, to avoid 3 × 3 windows, which include (according to the above conditions) at most only individual pixels. The maximum value of p consists in the length of the shorter side of image as well. For each scanning window that meets the above conditions, the system executes the following steps:

(1) Computation of the area of the identified dust, as the sum of the scanning window pixels.
(2) Initialization (or increase) of a counter, which takes into account the number of dust found for that area value.
(3) Determination of the shape of the found element, based on the area ratio, as indicated below.

After completing these three steps, the system flushes the scanning window, in order to stop the algorithm from working on an already examined dust element. Finally, the value of p is increased by one and the scan restarts. This occurs until the system analyses all the elements.

Regarding the area ratio, the system use a heuristic criterion to discriminate circular shapes from filiform shapes. In the case of granular dusts, the ratio between the area occupied by the dust and the area of the scanning window will be close to one; instead, area ratio will be lower in the case of the filiform dusts. This property is sufficient to discriminate between the two types. In particular, if we consider a scanning window of side p, the maximum area that can characterize a filiform dust results by:

(6) $$A_{\text{max}}=\left(p-2\right)\frac{p}{3}$$

In fact, a filiform dust can have maximum length of p−2 (because two pixels are dedicated to the edge of the window which, as said, must remain empty), while the width is fixed at 1/3 of the side of the window (to maintain a rangy look). Hence, the threshold value δ of the area ratio results as:

(7) $$\delta =\frac{A_{\text{max}}}{p^{2}}$$

**Figure 4.**
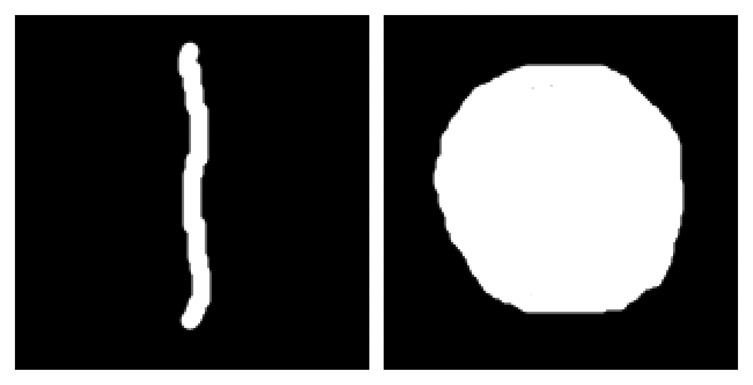
The two simplest dust types considered for the dusts classification: filiform and circular shapes.

For example, the dust elements show in Figure 4 present an area ratio of about 0.12 (filiform) and of about 0.50 (circular); in this case, the δ threshold is approximately equal to 0.32.

**Figure 5.**
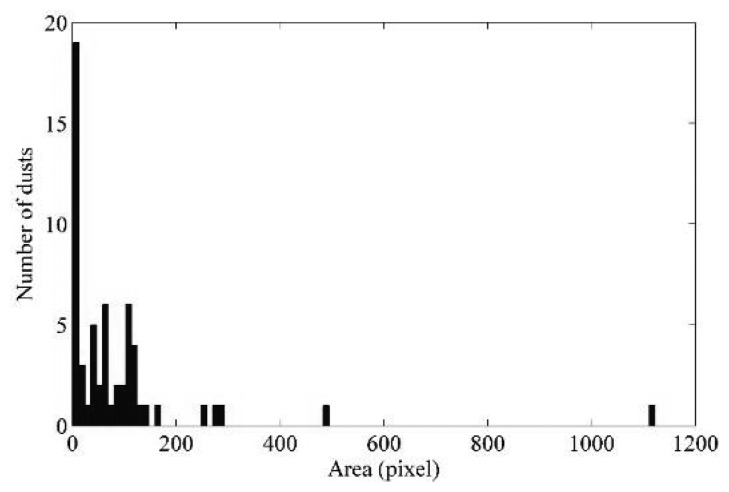
An example of a dust amplitude spectrum for a captured image.

The proposed approach aims to obtain the dimensional spectrum of the dust shown in each captured image, *i.e.*, an overview of how the overall number of individual dust occurs in terms of size. Figure 5 shows an example of dust amplitude spectrum.

Multiplying the dusts area in pixels by the value in µm^2^ of a single pixel, the system returns the dust area in µm^2^. The pixel size in µm^2^ varies according to the sensor used to capture the original images. In our case, the pixel area of the MT9V032 CMOS sensor is of 36 µm^2^ [38]. Moreover, the algorithm assigns a shape label to each dust element (selected between granular shape and filiform shape). Finally, it also returns two counters of how many granular and filiform elements are located in the image.

## 4. Tests and Results

**Figure 6.**
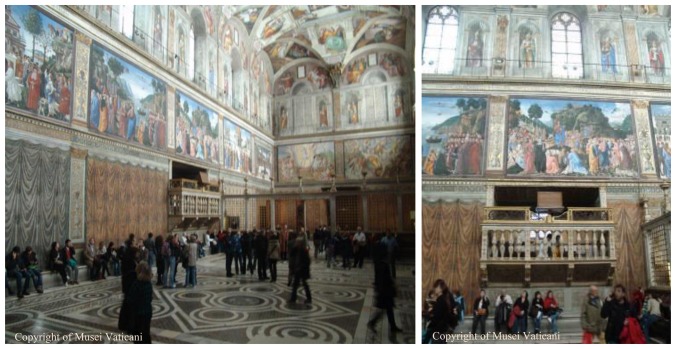
The “Cantoria” viewed from the Sistine Chapel.

Some experimental sessions took place in the Vatican Museums, in Rome, Italy, and in particular in the “Cantoria” (choir) of the Sistine Chapel (Figure 6), in order to test the proposed system for the analysis of dust accumulation. The artistic and scientific relevance of this test site was undoubtedly the main reason for our choice. As described in the following, the relevant flow of visitors affects the dust deposition mechanisms, making it particularly suited for our studies. In addition, this environment provides a 24 h Internet connection, which allows us a permanent remote accessibility even at night.

**Figure 7.**
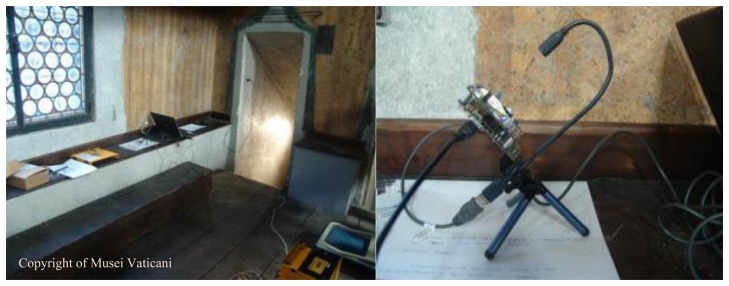
The acquisition device placed inside the “Cantoria” of the Sistine Chapel.

The “Cantoria” of the Sistine Chapel is an environment of about 6 m^2^ placed about 3 m up the floor of the chapel (Figure 6). This environment has a wooden seat, where we placed the sensor (Figure 7). A closed door did not allow entry to visitors. Thanks to the use of the previously mentioned LED lighting, the sensor was insensitive to sunlight, although there was a window facing on the outside.

**Figure 8.**
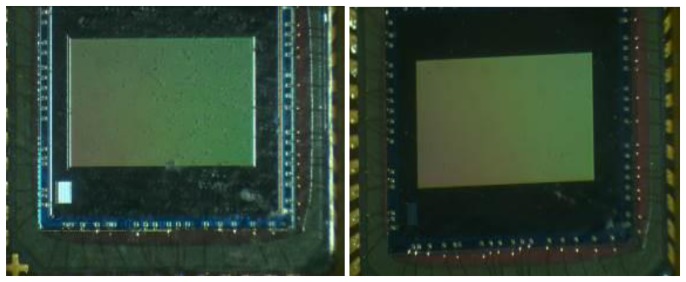
Comparison between the sensor before and after cleaning.

The sensor was placed with a 45° tilt angle (Figure 7). In this way, the dust will be unable to accumulate permanently (as might occur with a horizontal surface) and unable to immediately fall from the sensor (as might occur with a vertical surface). The acquisition time interval was 30 min, in order to have a high number of images to analyse. The acquisition session took two weeks, at the end of which the sensors provided about 750 images. Before its positioning, the sensor has been cleaned with compressed air, then with a soft-bristled brush and, finally, with a cleaning cloth for photographic lenses (see Figure 8).

The position of the Cantoria is above the level where the visitors normally walk, ensuring the absence of turbulence caused by the flux of persons and making the measurements stable. Choosing the Cantoria as the test site results in a logistic advantage due to the ability to exclude any interference and damage caused by visitors. In addition, since the Cantoria overlooks the Sistine Chapel through a direct connection, its environment is comparable to the Sistine Chapel, involving the same type of dust accumulation.

**Figure 9.**
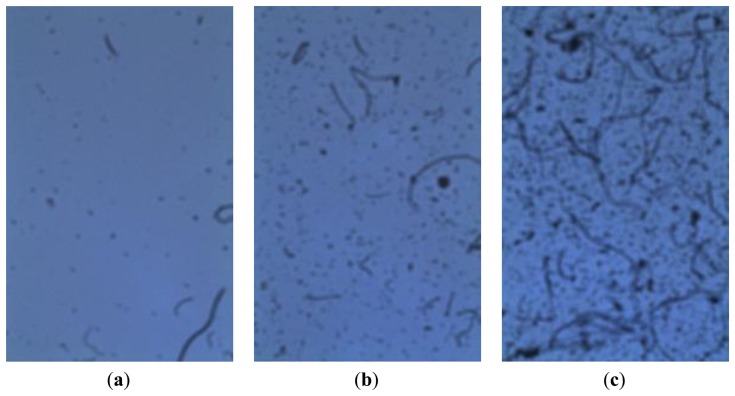
Some images acquired at different times.

The proposed approach was tested on some of the acquired images. Figure 9 shows three samples of the images used for testing. They represent three general study cases, as they show three completely different degrees of soiling.

As already said, the output of our algorithm consists of two different types of information: one related to the identification of the shape of dusts and one concerning their size. The first information consists of two counters, which enumerate both the circular and filiform dusts. In addition, the algorithm assigns the shape type to each individual dust, identifying it with a different colour (black for the circular type and grey for the filiform type). Moreover, the algorithm returns the dimensional spectrum of the dusts, evaluating the size associated with each dust element. At the end, the algorithm shows all the discarded elements, due to the limitation imposed by the maximum scanning window size (these elements can be analysed whit a larger scanning windows, which implies a longer computation time).

**Figure 10.**
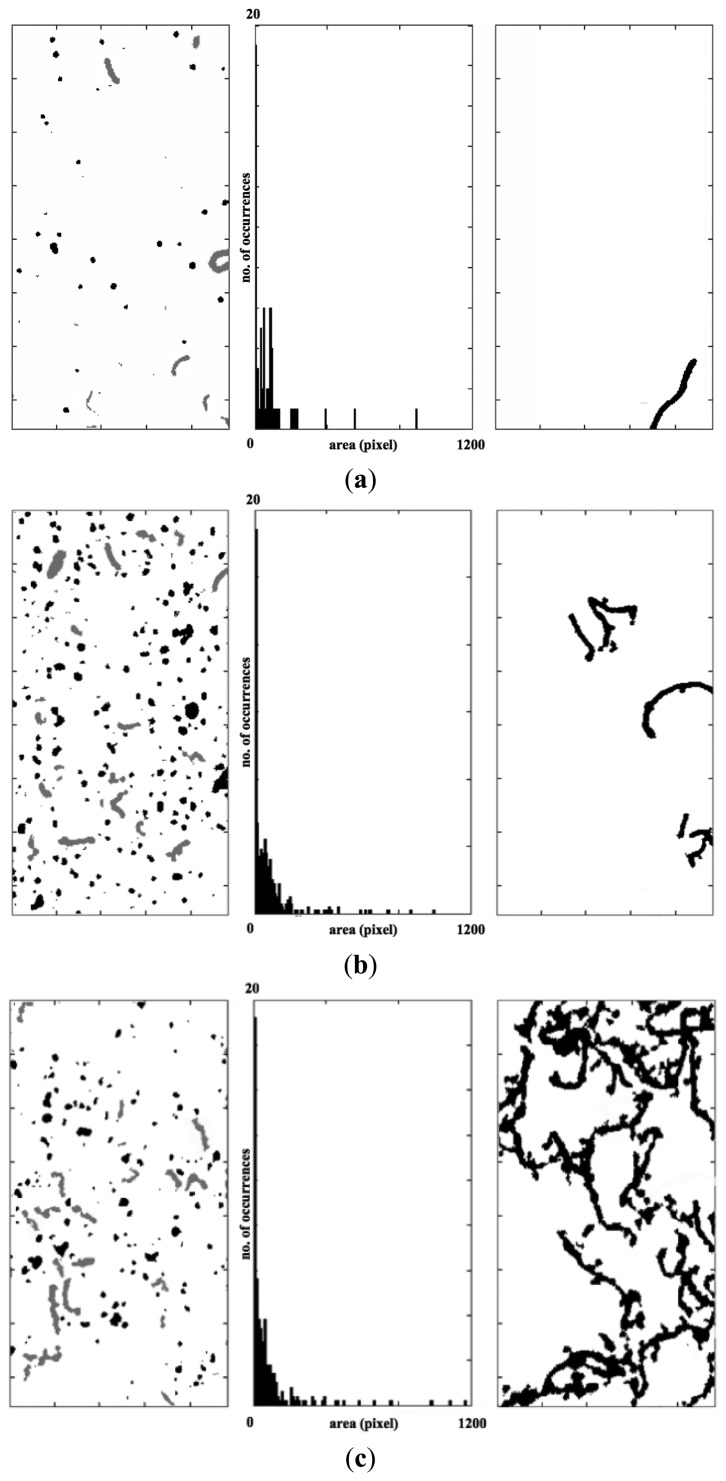
Output results for the three samples image: the dusts classification (black for the circular type and grey for the filiform type), the dusts amplitude spectrum and the discarded elements due to the scanning windows size.

Figure 10 shows the algorithm outputs related to the three sample images. As can be noted, the analysis is much more complex if the image shows many dusts (Figure 9c), due to the overlapping that can occur between two or more elements. This issue mainly depends on the low available focus, since the sensor is directly in contact with the powders. The application of erosion operators can overcome this problem. They can make the image thicker, separating some overlapping elements. As shown in the middle images of Figure 10, the amplitude spectrum provides quantitative information about the dust accumulation. In particular, the three spectrums show a very relevant peak corresponding to the finer dust. These contributions concern the soil dust, which area is typically lower than 350 µm. The middle components of the spectrum depend mainly on biological contributions (dandruff, skin debris, *etc.*) or anthropic activities (building works, maintenance, restoration, *etc.*). Finally, the larger elements are the fibres, which are textile (clothing) and biological (hair and fur). This classification results from an a priori optical microscope analysis.

**Figure 11.**
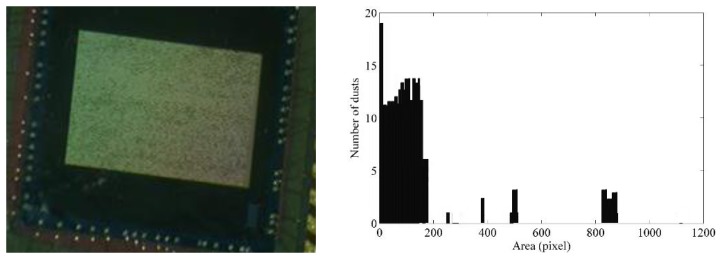
The sensor during the laboratory test and the resulting amplitude spectrum.

In order to compare the analysis within the Cantoria, we have performed an additional acquisition and processing using the proposed system within the Laboratory of Electrical and Electronic Measurements at the University of Roma Tre during painting and sanding works. This analysis showed a significant higher contribution of the elements of the middle portion of the spectrum, due to the mentioned works, as shown in Figure 11. In fact, the tests performed inside the Cantoria did not shown these contributions, since that type of work had not affected it in times closer to our analysis.

These information do not depend directly on the data concerning the visitors flow inside the experimentation site, especially because an essentially constant flow of visitors affects the Sistine Chapel during the opening, regardless of day and time. However, our acquisitions and processing provide interesting information about the mechanism of dust accumulation. In fact, immediately after the closing, the captured images show a rapid accumulation of dust elements. A few hours past midnight this accumulation stops and the captured images tend to remain unchanged. This stability changes when the Museum opens to visitors. Note that the pre-opening time does not show substantial changes, even though the ventilation plant start-ups and the safety check occurs, demonstrating how the visitor flow is the main source of movement and alteration of the deposits.

The performed tests show good performance in terms of execution time, which never exceeds 10 min (the tests were carried out on a standard machine, *i.e.*, x64 Intel Core i3 with 8 GB DDR3 RAM). Regarding the accuracy of the shapes recognition, the proposed method never drops below a value of correctness of 85%, compared to a standard labelling carried out by an expert.

## 5. Conclusions

The proposed system is able to recognize and assign a label concerning the shape of the single element (choosing between “filiform” and “circular”) and to evaluate the size of each element of the dust, returning the dimensional spectrum of the deposit. Moreover, the algorithm returns the degree of soiling of the image (expressed as a percentage), the number of elements having a circular shape and the number of elements having a filiform shape.

The most critical parameters of the proposed approach are the cut-off frequency of the low pass filter used for the subtraction of the background (in order to remove a brightness gradient) and the size of the scanning window (which affects not only the execution time, but also the number of non- processed elements). A study of a method for the automatic determination of these parameters was started in order to improve and refine its performance.

Some studies have been started on the possibility of associating also information on the chemical composition of the identified dust elements. This could be possible using the colour information (discarded in the proposed approach in favour of processing performance) and some objective observations resulting from chemical analysis carried out in advance (for the construction of reference classes that go beyond the simple distinction between filiform and circular). Moreover, a data mining analysis on the correlations between the flow of visitors and the results obtained from our system has been started, also involving more information about the Vatican Museum (building and outdoor location). In the next future, an array of sensors within the Sistine Chapel will allow drawing detailed correlations between the test environment and the experimental results, especially in terms of anthropic impact and of the efficiency of the air conditioning systems.

Finally, we are studying a different approach based on the use of computational intelligence techniques (such as classifiers and neural networks) to address the problem of shape recognition and classification of the individual elements of dust.
